# Atlas and Epitome of Human Histology and Microscopic Anatomy

**Published:** 1903-05

**Authors:** 


ATLAS AND EPITOME OF HUMAN HISTOLOGY AND MICROSCOPIC ANATOMY.—By Privatdocent Dr. J. Sobotta, of Wurzburg. Edited, with additions, by G. Carl Huber, M. D., Junior Professor of Anatomy and Histology, and Director of the Histological Laboratory, University of Michigan, Ann Arbor. With 214 colored figures on 80 plates, 68 text-illustrations, and 248 pages of text. Philadelphia and London: W. B. Saunders. 1903. Cloth, $4.50, net.

This work, like others of the same series, has a triple value: First, it is useful as an atlas; second, a text-book for students; and third, a book of reference for practitioners. It is admirably arranged and most excellently illustrated, the coloring so artistically applied as to bring out the characteristic features of each individual cell.

The subject-matter is concise and well chosen, being ample; reinforced as it is with self-explanatory diagrams, which is, after all, the most effectual way of teaching.

We strongly recommend the work. T. P. L.

